# Simplicity lacks robustness when projecting heat-health outcomes in a changing climate

**DOI:** 10.1038/s41467-020-19994-1

**Published:** 2020-11-27

**Authors:** Jennifer K. Vanos, Jane W. Baldwin, Ollie Jay, Kristie L. Ebi

**Affiliations:** 1 Arizona State University, School of Sustainability, Tempe, AZ USA; 2 Lamont-Doherty Earth Observatory, The Earth Institute, Columbia University, Palisades, NY USA; 3 The University of Sydney, Thermal Ergonomics Laboratory, Faculty of Medicine and Health, Sydney, NSW Australia; 4 The University of Sydney, Charles Perkins Centre, Sydney, NSW Australia; 5 Center for Health and the Global Environment, University of Washington, Seattle, WA USA

**Keywords:** Physiology, Climate sciences, Climate-change adaptation, Climate-change impacts, Climate-change mitigation

## Abstract

Extreme heat adversely affects human health, productivity, and well-being, with more frequent and intense heatwaves projected to increase exposures. However, current risk projections oversimplify critical inter-individual factors of human thermoregulation, resulting in unreliable and unrealistic estimates of future adverse health outcomes.

Extreme heat is a global health concern, and human exposure to dangerous heat is expected to increase with climate change and urbanization. While Earth system models incorporate substantial complexities of the climate system, current projections of heat-related illness or death do not adequately account for the intricacies of human physiological heat responses, which are critical for determining vulnerability to extreme heat. This mismatch leads to projections of future heat-related mortality, survivability, and liveability that are not realistic or robust.

Oversimplification of these complex responses, such as assuming that a single temperature value adequately predicts death, or using inappropriate heat stress metrics, can result in unrealistic projections of the range of future heat-related health outcomes, from well-being to illness to death. Discounting human adaptive capacity introduces further uncertainties. Consequently, decision-makers may be poorly informed about evolving heat-related risks, potentially resulting in under-preparation or over-spending of scarce health system resources.

Given the global threat of heat to human health, productivity, and well-being, there is an urgent need to provide more robust and realistic projections of future heat-related health risks to assess both survivability and liveability in a warming world. This assessment requires combining knowledge from climate science, physiology, and epidemiology. Modeling advancements that accurately characterize the range of human responses to extreme heat can provide more robust climate impact assessments to enhance policy decisions and interventions that protect the most vulnerable people from current and future impacts of extreme heat.

## Cascade of factors leading to heat stress, strain, and adverse health outcomes

Heat stress is defined by the net heat load imposed on a person from the combined thermal effects of the environment (air temperature, radiant temperature, humidity, and wind), activity (metabolic heat production), and clothing. The resultant heat strain is physiologically characterized by the associated rise in body temperature due to body heat storage, dehydration from sweat losses that are not replenished, and an increase in cardiovascular strain as heart rate rises to maintain blood pressure in the face of peripheral vasodilation^1^. The observed strain for a given level of heat stress is dependent on the physiological capacity of a person to modify skin surface heat dissipation through sweating and vasodilation. As such, there is wide inter-individual variability of the physiological human heat stress response, resulting in different experiences of heat strain even under similar environmental conditions.

The quantity of heat accumulated inside the body over time ultimately determines the rise in body temperature. Therefore, the duration of heat exposure is a key, but not the only, factor when considering the risk of heat stress and strain. The temporal duration of exposure and temporal resolution of environmental data that are relevant to predict a given exposure depend on the type of outcome (e.g., classic versus exertional heat stroke) and the population under consideration. For example, understanding future exertional heatstroke in bricklayers in India requires time-of-day information in sub-hourly timescales, yet projecting classic heatstroke to the end-of-century would optimally use 24-h data to apply existing epidemiological models. In many populations, individuals frequently adjust activity and behavior, such as slowing, stopping, and/or seeking refuge before heat strain begins to feel intolerable, thus necessitating very high temporal resolution exposure data (i.e., 15-min time steps) to accurately model heat stress or strain. However, lower temporal resolution data may be sufficient for more vulnerable populations (e.g., older adults) as their activity levels are typically light and behavioral capacity to alter exposure is limited). In these populations, body temperature rises slowly and classic heat stress gradually manifests over days. Therefore, sufficiently long exposure durations must be considered when forecasting heatstroke risk. When climate models output average environmental conditions over, for example, 6-h increments, the exposure should be integrated over multiple 6-h windows to accurately estimate classic heat stroke. Overall, the duration relevant to human health depends on the question, the population, and the type of health outcome.

The risk of a prevailing level of heat strain leading to adverse health outcomes, such as heat exhaustion, heatstroke, cardiovascular collapse, or renal failure is much higher in people with pre-existing illness (e.g., immunocompromised^2^, cardiovascular disease^3^). Emerging evidence also indicates a higher risk of stillbirth in pregnant women exposed to extreme heat^4^. Factors such as age^5^, certain medications or drugs^6^, and body composition^7^ may exacerbate heat strain due to sweating and/or vasodilatory impairments, whereas aerobic training^8^, acclimatization, and behavioral^9^ adaptations can be protective. The productivity and health of outdoor workers and people engaging in sports/recreation can also be heavily impacted by extreme heat. To confidently forecast a location as ‘survivable’ in the future, it must be so across all stages of life. Moreover, for meaningful human activity to occur in a given location, it must also be liveable whereby the climate can safely sustain work and play for an extended period.

Capturing these complexities allows researchers to understand the level of heat strain that eventuates from heat stress, and by extension heat-related health outcomes. These complexities are currently neglected within common heat-related health projections. For example, the most commonly used metric for projecting future heat-related mortality is the wet-bulb temperature (*T*_w_) threshold of 35 °C (e.g.^10^,), which is based on a thermodynamic limit to heat exchange whereby the human body becomes an adiabatic system (Table 1). The conservative assumption that this value must be reached to cause widespread death is only valid under a specific set of conditions, i.e., the person is completely sedentary, unclothed, maximally heat acclimatized, and an average-sized adult free from any thermoregulatory impairments. These assumptions are implausible in the real-world, and severe illness and death can occur at much lower heat stress levels when considering realistic metabolic heat loads, clothing, population demographics, and health status. In essence, using this *T*_w_ threshold without questioning such implicit assumptions could result in substantial underestimation of the future range and potential severity in heat-related outcomes. Conversely, the single threshold can also overestimate risk as humans are known to live in harsh climates through buffering the effects of climate extremes using adaptive innovations. Often these innovations involve technological, infrastructural, and behavioral adaptations that support minimizing extreme exposures and/or the amount of time an individual is exposed to the given extreme^11^.

**Table 1:** Heat metrics or thermal indices often used within climate model projections in recent studies.

Metric	Description, application, and notes on use
Psychometric wet bulb temperature (*T*_pwb_)	•Temperature of a parcel of air that is cooled to saturation by the evaporation of water into the air, with the latent heat for evaporation supplied by the parcel^20^ •Also called the thermodynamic wet bulb temperature •When *T*_pwb_ approaches skin temperature, all heat loss avenues are eliminated, thus net heat dissipation is zero •Assumes the human body becomes an adiabatic system
Wet bulb globe temperature (WBGT)	•Indicator of heat stress on the active/working body in direct sunlight •WBGT = 0.7 *T*_nwb_ + 0.2 *T*_g_ + 0.1 *T*_db_, where *T*_nwb_ is non-aspirated, “natural” wet bulb, *T*_g_ is black globe temperature, *T*_db_ is shaded dry bulb (air) temperature •Intended for use in active populations outdoors; developed for military from studies in hot, humid environments •Studies, particularly climate projections, often neglect the *T*_g_ value, which is not its intended use
sWBGT (simplified WBGT)	•sWBGT = 0.567*T*_db_ + 0.393*e* + 3.94; *e* = vapor pressure •Approximation does not account for variations in the intensity of radiation or wind speed, yet assumes a moderately high radiation level in light wind conditions^21^ •May lead to overestimates of thermal stress in windy and cloudy conditions or underestimates of thermal stress in dry, sunny, hot conditions when required sweat rates are high due to activity levels
Apparent temperature (AT)	•An adjustment to the ambient temperature based on the level of humidity for a typical human, which sometimes incorporates solar radiation •Derived from human heat balance principles •vVarious formulas exist to approximate AT, many of which ignore radiation
Heat index	•A simple hot weather version of the AT to describe a ‘feels like’ temperature •Uses multiple regression of temperature and relative humidity based on original AT (above) •Over 21 approximations exist

## Quantifying uncertainties in climate and health projections

**Fig. 1:**
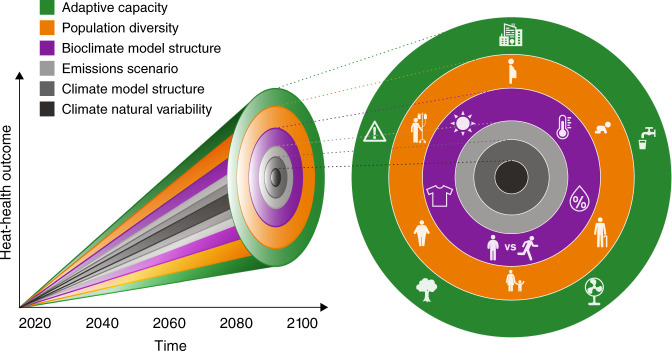
Uncertainties in projections of human health, well-being, and productivity due to extreme heat exposure in a warming climate. The left side of the figure shows that in addition to uncertainties regularly quantified in projecting environmental variables (climate variability, model structure, and emissions scenario), there are further uncertainties in projecting heat-health outcomes including bioclimate model structure (examples in Table 1), diversity and vulnerabilities in the population, and various adaptations to heat (e.g., warning systems, behavior, urban planning). This plot is intended to be illustrative rather than quantitative—the respective magnitude of these additional sources of uncertainty remains unknown. The right side provides graphics of these sources of uncertainty in projecting human heat-health outcomes, with the concentric circle colors corresponding with the colors of uncertainty cones in the left side. These graphics represent inputs to bioclimate models, including solar radiation, temperature, humidity, clothing, and activity; considerations for population diversity, such as pregnancy, age, weight, and pre-existing illness; and various forms of adaptive capacity, such as building design, hydration, fans or air conditioning, green infrastructure, and the implementation of heat warnings systems.

Climate change projections employ sophisticated methods for addressing three main sources of uncertainty when projecting environmental variables:^11^ uncertain future emissions of greenhouse gases and aerosols, limitations in scientific understanding of the climate system, and difficultly predicting natural variations of the climate system. Each source of uncertainty is quantified differently. Emissions scenario uncertainty is addressed by running climate models with various trajectories of future emissions (e.g., Representative Concentration Pathways) that reflect the range of development and policy trajectories human society may follow. Uncertainty due to limitations in scientific understanding (called structural uncertainty) is assessed utilizing an ensemble of different climate models (e.g., Coupled Model Inter-comparison Projects) that reflect different assumptions about atmosphere, ocean, and land processes. Finally, uncertainty due to natural variability of the climate system is assessed by running many climate projections with slightly different starting conditions. Natural chaos in the system leads each simulation to follow a different trajectory, facilitating a sampling of the range of natural variability. These uncertainty sources vary in magnitude and relative importance moving into the future (Fig. 1) yet are key to accurately assess the risk of climate-related hazards, and in turn, adaptation, decision-making, and preparedness.

Analogous to the assessment of multiple types of uncertainty in climate projections, we propose developing frameworks to quantify uncertainty in human physiological and behavioral factors in future heat-related health outcomes modelling. These frameworks require accounting for the cascading flow from weather conditions and potential heat exposure to heat stress, heat strain, and finally adverse human health outcomes, noting that survivable outcomes may not be liveable. As displayed in Fig. 1, three sources of uncertainty are present in this cascade: population diversity, including health and socioeconomic status, cultural norms, occupation, and age; adaptive capacity; and the structure of the chosen bioclimate model. These sources of uncertainty can interact to increase or decrease vulnerability. For example, low-income populations may have greater constraints in altering their environment or behavior (such as work intensity) and may suffer from pre-existing health concerns that exacerbate heat-related risks. In practice, accounting for population diversity and the capacity of individuals and organizations to manage increases in extreme temperatures would inform developing and implementing adaptation strategies that focus on the most vulnerable across the full range of adverse health outcomes.

### Uncertainties related to bioclimate models

Bioclimate models or thermal metrics attempt to predict aspects of human thermal comfort, stress, or strain through integrating weather variables with human factors (activity and/or clothing). While higher temperatures, humidity, and radiation increase heat strain, there is substantial debate on how best to quantitatively model human physiological responses to heat and with what metric or bioclimate index. Among the >160 thermal metrics available, only simple metrics have been used for projecting heat-related mortality (Table 1), which we hypothesize is due to a lack of communication between climate and health scientists and the straightforward nature and presumed physical intuitiveness of the simple indices.

These simple metrics each have strengths and weaknesses that are directly comparable to climate model structural uncertainty. Yet each metric or index may provide a wildly different outcome that is highly dependent on how and why the given index was created and its application. For example, the WBGT––developed to manage heat illness during military training in hot, humid environments^12^ and later adapted for other occupational and sports settings––has limited integration of human physiology and cannot directly predict physiological heat strain. Conversely, heat indices based on human heat balance can incorporate known physiological and behavioral modifiers of heat dissipation (e.g., predicted heat strain or UTCI-Fiala models). These advanced models utilize heat imbalance rates over time to predict indicators of physiological strain (e.g., core temperature change, sweating required), and thus determine the environmental limits that a particular individual can tolerate for a given combination of activity and clothing.

### Uncertainties related to population-level heat adaptation

Heat mortality projections must confront uncertainties in vulnerabilities in present and future population distributions. Considerations include population growth and aging, how the burden of cardiorespiratory diseases could evolve, the extent of future adaptation, and how cultural norms could shift. Using exposure-response relationships developed primarily in temperate regions (generally with higher income and with adaptations such as higher prevalence of air conditioning) are unlikely to accurately estimate the risks associated with the higher temperatures in the tropics^13^.

There is significant uncertainty in future adaptation to changing heat-related hazards and whether current models can be appropriately applied to conditions outside of historic experience^14^. Recent heatwaves, for example in Japan and Sweden^15,16^, brought temperatures far outside the historic range, indicating that adaptation to current heat events may be insufficient for future events^17^. In some but not all regions, there is evidence that individuals are behaviorally adapting to higher temperatures, presumably because of increased access to air conditioning^16^ and/or greater use of evaporative cooling with fans^18^. Early warning and response systems can also reduce mortality through raising awareness of heat-related risks, including steps to reduce exposure (e.g., cooling centers)^17^.

Grappling with these adaptation uncertainties across cities is critical to understanding the level of health risks posed by climate change^19^, and a standard set of adaptation scenarios would enhance the comparability of studies and articulate the relevant health impacts. Despite the utility of more complex human models, they are yet to be applied to create an ‘ensemble’ (or a group of different pathways) of potential human responses to future extreme heat to inform adaptation options (Fig. 1). For example, each pathway could incorporate the additional considerations of the human body, as well as differing responses, behavior, and clothing, among other physiological attributes, that can either help or hinder one’s ability to manage high heat exposures. These considerations broaden the range potential of plausible heat-related health outcomes, as shown by the green and orange colored pathways in Fig. 1.

## Conclusions

Heat-related morbidity and mortality are preventable in most circumstances with awareness and access to supportive infrastructure. Robust projections of future heat-related health risks are needed to assess adaptation needs, survivability, and liveability in a warming world. Achieving this goal must move beyond the consideration of potentially hazardous environmental conditions to address the cascade of physiological and behavioral factors that ultimately determine health. Using an ensemble approach not only for climate, but also for human factors related to behavior and physiology, as well as expanding studies to consider low- and middle-income countries, will improve the robustness and reliability of projections of heat strain and health concerns.

The general applicability, and thus usability, of projections are questionable when they are based on a single ambient threshold at which mortality is presumed to occur applied to an inanimate unclothed human (e.g., *T*_w_ of 35 °C) versus a range of outcomes with underlying uncertainties. Moreover, without considering the temporal duration of exposure, space, activity, clothing, behavior, and most of all, individual physiology, the mismatch between complex climate models and over-simplified human models fails to provide useful information for decision-makers. Embedding more sophisticated human heat stress models into climate projections would provide relevant health projections across a more realistic and therefore diverse population than an assumed idealized individual.

In practice, this information could shift efforts towards the appropriate provision of heat adaptation strategies and management of heat exposure for the most vulnerable populations; more appropriate management of scarce resources; effective adaptation strategies and heat-aware urban growth; and underline the need for climate change mitigation. Without collaboration between the climate and health science communities, future heat risks will continue to miss large portions of the population either above or below the current one-size-fits-all approach, potentially leading to ineffective mitigation and adaptation policies.
